# Rapid Molecular Identification of Scolytinae (*Coleoptera*: *Curculionidae*)

**DOI:** 10.3390/ijms20235944

**Published:** 2019-11-26

**Authors:** Jonathan Eric Albo, Jean-Philippe Marelli, Alina Sandra Puig

**Affiliations:** 1 Subtropical Horticulture Research Station, USDA-ARS, Miami, FL 33158, USA; jea16@my.fsu.edu; 2 Mars/USDA Cocoa Laboratory, Miami, FL 33158, USA; jean-philippe.marelli@effem.com

**Keywords:** DNA barcoding, *Coptoborus*, *Xyleborus*, *Theoborus*, *Hypothenemus*, *Araptus*, molecular markers, ambrosia beetles, bark beetles, Scolytinae

## Abstract

Routine identification of bark and ambrosia beetles is done using morphology. For people lacking the necessary taxonomic knowledge, proper identification of a novel specimen can be challenging and time consuming. This study compares the usefulness of four genetic markers (28S, EF-1a, ITS2, and COI) and five primer pairs (D2F1/D3R2, eflafor1/eflarev1, ets149/efa754, ITS2F/ITS2R, and LCO1490/HCO2198) to identify Scolytinae beetles, and outlines a molecular identification strategy, with results possible in two days. Markers COI and EF-1a were selected based on the ability of the respective primers to amplify DNA from multiple genera (*Coptoborus*, *Xyleborus*, *Hypothenemus*, *Theoborus*, and *Araptus*) and the ability of the resulting sequences to provide accurate and unambiguous matches in GenBank. BLASTn analysis of EF-1a sequences (both primer pairs) correctly identified four out of the five genera and COI sequences identified at least one sample of every genus tested and was the only primer pair to correctly identify *Araptus* specimens. Further, 28S sequences successfully identified *Coptoborus, Xyleborus*, and *Theoborus* but not *Hypothenemus* or *Araptus*. The low number of EF-1a (1), 28S (7), and ITS2 (0) sequences from *Araptus* individuals present in GenBank compared with COI (137) is likely the reason that only the latter marker was capable of identifying members of this genus. ITS2 sequences were insufficient to identify any of the samples tested. This study also determined the minimum quantity of DNA that could be used for molecular identification. Primers D2F1 and D3R2, which had the highest rate of amplification in all genera tested, could yield an informative sequence with as little as 0.00048 ng of DNA, however, at least 0.0024 ng was needed for reliable amplification.

## 1. Introduction

Diseases associated with wood-boring ambrosia and bark beetles (Curculionidae: Scolytinae and Platypodinae) are a rapidly increasing threat to forests and fruit production around the world [1,2]. The accumulation of new introductions has been exponential, which is reflected in published literature [3,4]. Over half of all journal articles on this taxonomic group have been published within the past ten years [1]. Some of these pathosystems, such as Dutch elm disease [5], have been the focus of research for many years, but others such as laurel wilt vectored by the redbay ambrosia beetle (*Xyleborus glabratus*) [6,7], thousand cankers disease caused by the walnut twig beetle (*Pityophthorus juglandis*) [8], and *Fusarium* dieback spread by shot hole borers in the *Euwallacea fornicatus* species complex [9,10] are the result of pest introductions that have prompted more recent investigations.

Although Scolytinae represent over half of all insects intercepted at U.S. ports of entry [11] and 93% of insects on solid wood packing materials [12,13], only 40% of these were identified to the species level [14]. A molecular identification approach that could be performed using basic molecular biology skills and equipment could address this knowledge gap.

Although genetic sequences of these insects have been generated from diversity analyses and entomological studies [15,16], molecular identification of ambrosia and bark beetle species is not used on a wide scale. In addition, this is the only way to make reliable identifications from structurally damaged samples where key morphological characteristics are absent or compromised, or to distinguish among members of a cryptic species complex (e.g., *Euwallacea* nr. *fornicatus* [17]).

To develop an efficient introductory approach for molecular identification of ambrosia and bark beetles, this study examined three DNA extraction protocols, determined the minimum amount of DNA that could yield an informative sequence, and compared the usefulness of five primer pairs in distinguishing several genera. These methods can be used by plant pathologists, horticulturalists, and agricultural inspectors lacking specialized knowledge of insect taxonomy.

## 2. Results

### 2.1. Quantity and Quality of Extracted DNA

**Figure 1:**
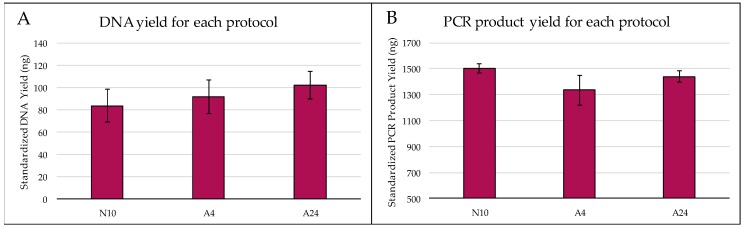
Evaluating different DNA extraction protocols, N10, A4 and A24, which differ based on lysis time and method of grinding. (**A**) No statistically significant differences in mean (± SE) standardized DNA yield were found among protocols. (**B**) Mean yield (± SE) of PCR product following. DNA amplification with primer pair D2F1/D3R2 was measured to evaluate quality of DNA extracted with each protocol. No statistically significant differences were found among extraction protocols.

Concentrations of *Theoborus* sp. DNA obtained with different extraction protocols ranged from 0.91 to 3.44 ng/μL (Figure 1). A24 had the highest mean (± SE) standardized DNA yield (102.29 ng ± 12.36), followed by A4 (92.05 ng ± 15.26), then N10 (83.75 ng ± 14.69). However, these differences were not statistically significant (*F* = 0.45, df = 2, *p* = 0.644).

Similar levels of DNA quality (measured as PCR product yield from 1.5 ng DNA) were obtained with the different extraction protocols (Figure 1B). N10 had the highest PCR product yield (± SE) (1502 ng ± 38), followed by A24 (1440 ng ± 41.8), then A4 (1334 ng ± 117). However, since these differences were not statistically significant (*F* = 1.31, df = 2, *p* = 0.298), N10 was used for all subsequent DNA extractions as it was the least time consuming (~1 h).

### 2.2. DNA Threshold for Molecular Identification Using D2F1/D3R2

**Figure 2:**
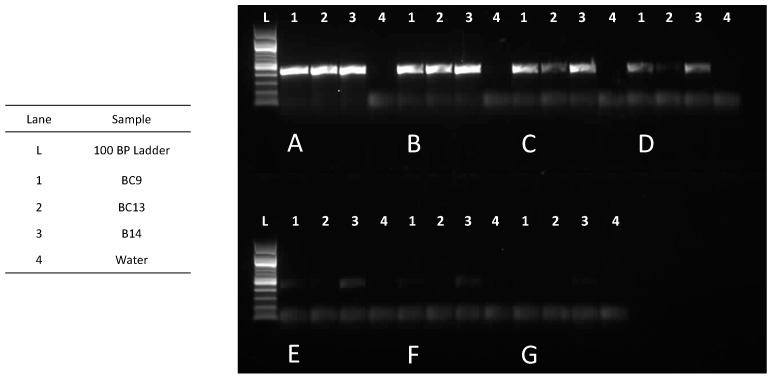
Amplification of decreasing amounts of DNA from three *Theoborus* sp. beetles using primers D2F1 and D3R2 (1% agarose gel *w*/*v*) was performed to determine the smallest quantity of DNA that could still be successfully amplified. DNA quantity started at (**A**) 1.5 ng and was decreased by a factor of five each time. Subsequent levels had (**B**) 0.3 ng, (**C**) 0.06 ng, (**D**) 0.012 ng, (**E**) 0.0024 ng, (**F**) 0.00048 ng, and (**G**) 0.000096 ng of DNA.

At 1.5 and 0.3 ng, all samples showed clear amplification, and the representative of each that was sequenced (positive control), yielded high-quality sequences for the three tested *Theoborus* sp. beetles, B14, BC9, and BC13 (94.4%, 301 bp; 97.8%, 367 bp; and 99.3%, 299 bp, respectively) that had matches > 99.7% in GenBank to *Theoborus* sp. *B* (HM099718). At 0.06 ng, all three samples amplified but B13, which produced a faint band and yielded a lower quality sequence (90.4%, 291 bp vs. 97.4%, 423 bp, and 97.2%, 424 bp). Faint bands developed for all samples at 0.012 ng, and despite low PCR product concentrations (8.21, 7.16, and 3.12 ng/μL), they were sequenced and the first two samples had good matches (99.7%) with the correct genus (*Theoborus* sp.), as determined by the sequences obtained from the higher template samples. The third sample that amplified at 0.012 ng yielded a low-quality sequence (36%) that was 95% identical to two sequences of *Theoborus* sp. that differed by one base pair (HM099719, HM099718). B14 and BC9 produced faint bands at 0.0024 (PCR product concentrations of 4.20 and 2.66 ng/μL, respectively) and 0.00048 ng (PCR concentrations of 3.45 and 2.88 ng/μL, respectively), all of which yielded accurate matches in GenBank (>97%). At the lowest concentration of 0.000096 ng, B14 produced a faint band (3 ng/μL) but the resulting sequence was too low in quality (1.7–3.6% quality) to be informative (Figure 2).

### 2.3. Primer Pairs for Molecular Identification of Diverse Genera

**Figure 3:**
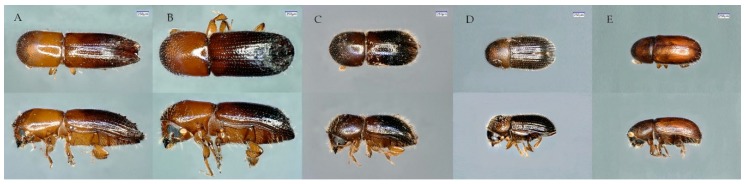
Representatives from the five genera tested in this study: (**A**) *Coptoborus*, (**B**) *Xyleborus*, (**C**) *Theoborus*, (**D**) *Hypothenemus*, and (**E**) *Araptus.* Size bar is representative of 250 μm.

The usefulness of primers D2F1/D3R2, eflafor1/eflarev1, ets149/efa754, ITS2F/ITS2R, and LCO1490/HCO2198 (Table 1) for identification of beetles in the *Coptoborus*, *Xyleborus*, *Theoborus*, *Hypothenemus*, and *Araptus* genera (Figure 3), is quantified as the proportion of each that amplified with a given primer pair, and the proportion that yielded a correct species or genus match following BLASTn analysis with the resulting sequence (Table 2). Primer pair D2F1/D3R2 amplified all samples except B20 (*Hypothenemus* sp.) and provided high-quality sequences (91.9% quality, 420 bp length) even with PCR product concentrations as low as 2.3 ng/μL (B16). However, sequences from *Hypothenemus* and *Araptus* isolates returned incorrect matches in GenBank (Table 3). The *Hypothenemus* isolates were 99.7% identical to sequences of *Theoborus* sp., with no *Hypothenemus* sp. accessions within the 100 best matches. There was only one 28S sequence from an *Araptus* specimen in GenBank but it had a lower % identity (87%) with the generated sequences.

Elongation factor sequences were capable of identifying all groups except *Araptus* sp. Primer pair eflafor1/eflarev1 amplified in all genera tested and yielded high-quality sequences (80–97%) (Table 4). However, they did not unambiguously identify members of the *Araptus* genus, with 91.6% identity with *Phelloterus* sp. (KY805860) vs. only 89.6% and 89.7% for *Araptus costaricensis* (KY852964) and *A. attenuatus* (FJ347565), the best matches within this genus. Primer pair ets149/efa754 amplified in all genera except *Araptus*, but yielded fewer high-quality sequences in *Theoborus* and *Coptoborus*, compared to the previous primer pair. For *Hypothenemus* individuals, two of the four samples generated sequences with quality scores above 89% and were at least 98% identical to *Hypothenemus* nr. *eruditus* sp. (JX264092) (Table 5). One of the four produced a low-quality sequence (26%) which shared 85.5% identity to *Phloeocleptus cristatus* sp. (KY805879) but this is not included in the results table due to the low quality of the input sequence.

Primer pair ITS2F/ITS2R amplified well in *Xyleborus* and *Coptoborus* and produced decent quality sequences (85.2–97.7%); however, a BLASTn analysis showed these sequences to be uninformative. Sequences in both groups (samples B7b and B4) were 100% identical to *Pityogenes chalcographus* (JQ066311) and *Galapaganus* spp. (EU748796) (Table 6), which are morphologically distinct from the samples processed as seen in the microscopy images for each of these samples. In addition, sequences obtained from two additional *Coptoborus* samples yielded no significant similarities for any matches in GenBank regardless of their qualities being < 97% and having lengths of 299bp and 679 bp.

Primer pair LCO1490/HCO2198 amplified well in all groups tested, but only about half of these resulted in good quality sequences (defined as quality greater than 40% by authors). For *Xyleborus*, *Theoborus*, *Hypothenemus*, and *Araptus*, the best sequence matches in GenBank were from individuals within the correct genus. However, identification of *Coptoborus* was ambiguous as the generated sequences had greater % identity to *Coptodryas* sp. (HM064072) than to accessions of the correct genus, which had a maximum % identity of 85.5% (Table 7). All sequences generated in this study are in Table S3.

## 3. Discussion

### 3.1. Quantity and Quality of Extracted DNA

DNA was extracted using the DNeasy Blood and Tissue Kit (Qiagen, Germantown, MD, USA) [18,19,23] using both the animal tissue protocol (Animal Tissue Protocol, DNeasy Blood and Tissue Handbook, Jul. 2006) and a supplementary insect protocol (Insect Protocol DY14, August 2006). The protocols differed primarily based on lysis time using a low and high lysis time for the animal tissue protocol and the suggested lysis time for the insect protocol (Table S1). Although lysis time and tissue disruption (such as grinding) can cause fragmentation and affect yield, there were no differences detected between the different protocols. Thus, it was determined that the most useful method was the insect protocol due to the rapid extraction. This protocol allowed the extracted DNA to be used downstream within the same day instead of over the span of the next several days.

### 3.2. DNA Threshold for Molecular Identification Using D2F1/D3R2

In this study, the minimum quantity of DNA required for reliable molecular identification with primers D2F1 and D3R2 was 0.0024 ng. This is well below the average concentration of all specimens extracted in this study (1.92 ng/μL), meaning that standard PCR protocols containing 1 to 1.5 μL of DNA can still be followed. Due to the presence of inhibitors in extracted DNA, volumes greater than 2 μL of DNA are rarely used in PCR. In published literature, 2–5 ng of template DNA were commonly used to obtain molecular sequences with Scolytinae [24,25]. Although some studies combined multiple individuals in a single DNA extraction to obtain up to 60 ng of template DNA [26], it is difficult for non-experts to reliably determine which beetles belong to the same species. Even beetles excavated from a single tree can belong to more than one species [27]. Since very small quantities of DNA can be extracted from a single ambrosia or bark beetle, it is valuable to know a reasonable minimum threshold.

### 3.3. Primer Pairs for Molecular Identification of Diverse Genera

Markers COI and EF-1a are recommended for initial molecular identification attempts based on the ability of the respective primers to amplify DNA from multiple genera (*Coptoborus*, *Xyleborus*, *Hypothenemus*, *Theoborus*, and *Araptus*) and the ability of the resulting sequences to provide accurate and unambiguous matches in GenBank. BLASTn analysis of EF-1a sequences correctly identified four out of the five genera and COI sequences identified at least one sample of every genus tested and was the only primer pair to correctly identify *Araptus* specimens. The low number of EF-1a (1), 28S (7), and ITS2 (0) sequences from *Araptus* individuals present in GenBank compared with COI (137) is likely the reason the latter marker was the only one capable of identifying members of this genus.

Primer pair D2F1/D3R2 amplified the best and generated high-quality sequences capable of identifying *Coptoborus, Xyleborus*, and *Theoborus*, however, neither bark beetle genus (*Hypothenemus* or *Araptus*) could be identified using these primers. Although six 28S sequences from *Hypothenemus* are available in GenBank, no *Hypothenemus* sp. accessions were within the 100 best matches. A single 28S sequence from *Araptus* sp. is available in GenBank (AF375297); however, 87% of the identity it shares with the sequences generated in this study is lower than the 91–93% shared with *Ips duplicatus* accessions. This may be due to misidentification, or high variability in the genus. Other *Araptus* species may have greater homology with the specimens in this study. As more sequences from confirmed *Araptus* species are deposited in GenBank, molecular sequence identification may become viable for members of this genus. Due to inconclusive results following BLASTn analyses, identification of J1, J2, and J4 was performed by Sarah Smith (personal communication, 28 October 2019).

The COI gene is considered the ideal gene for species identification of insects [28] due to its popularity and variety of different samples in GenBank. Sequences generated in this study based on the COI gene led to correct genus level identifications for all five genera analyzed. However, only five published studies were found where primer pair LCO1490/HCO2198 was used for Scolytinae [20,24,25,29,30]. These primers showed inconsistent amplification and low-quality sequences were generated in this study (even with high concentrations of amplified PCR product), indicating that LCO1490/HCO2198 may not be the best primer pair for amplifying COI in these organisms. Primers developed by Simon et al. [31] have been used successfully in Scolytinae beetles and may be a good alternative.

ITS2 sequences were insufficient to identify any of the samples tested, BLASTn results showed all sequence matches having query coverages of less than 50% with three out of the four sequences having a query coverage of less than 30% which may be due to low representation in GenBank. Additionally, it was difficult to obtain amplification for a wide variety of Scolytinae.

## 4. Materials and Methods

### 4.1. Insect Specimens

Adults of *Coptoborus*, *Xyleborus*, *Theoborus*, *Hypothenemus*, and *Araptus* were collected from diseased pods and stems of *Theobroma cacao* in Ecuador and Brazil. They were collected February–June 2019, stored immediately in 90% ethanol, which was decanted prior to shipping to USDA-ARS Plant Pathology laboratory in Miami, FL for molecular identification. All specimens were stored in 90% ethanol at room temperature (22–25 °C) and processed within 6 months of collection. Specimens were photographed with a Keyence VHX-5000 digital microscope (Keyence, Osaka, Japan) to calculate body length and to enable validation of resulting sequence identification. GenBank matches were based on max score following BLASTn analysis.

### 4.2. DNA Extraction

To determine the most efficient method for isolating DNA from ambrosia and bark beetles, three extraction protocols provided by Qiagen DNeasy Blood Tissue kit were tested on 19 whole beetle specimens of *Theoborus* sp. collected in Bahia, Brazil. N10 is the insect DNA extraction protocol provided as a supplemental protocol from Qiagen (Insect Protocol DY14, Aug. 2006) and includes the shortest lysis time (10 min) of the three methods. A4 and A24 follow the animal DNA extraction protocol described in the Qiagen DNeasy Blood and Tissue Handbook (Animal Tissue Protocol, Jul. 2006), with lysis times of 4 and 24 h, respectively. For the final resuspension, the volume of elution buffer (buffer AE) was decreased to 50 µL to account for the small amount of starting material. All samples were ground in 1× PBS or lysis buffer (buffer ATL), depending on the protocol, using a plastic pestle (DWK Life Sciences Millville, NJ, USA) in a 1.5 µL micro centrifuge tube. All centrifugation steps were carried out at room temperature (22–25 °C). The differences among protocols are summarized in Table S1.

**Figure 4:**
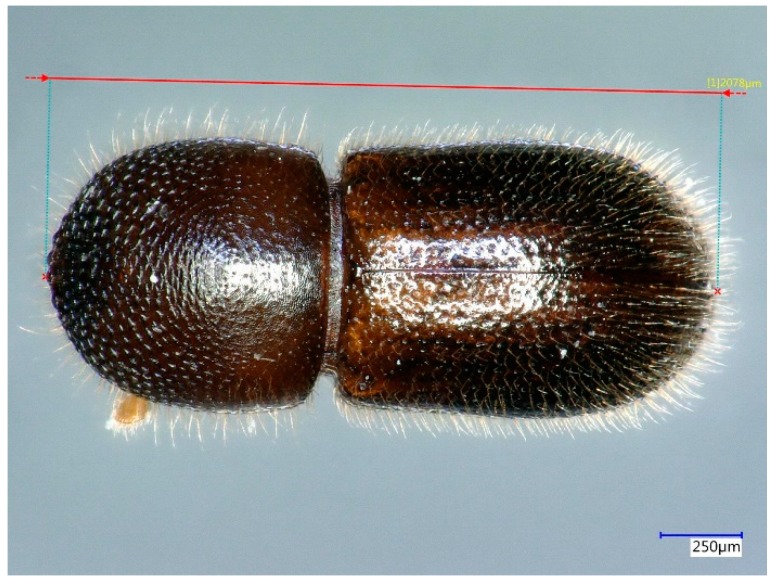
To compare extraction protocols DNA yield was standardized per specimen by dividing yield by body length then multiplied by 2000 µm. Specimen BC2 (extracted using protocol A24) is displayed here to illustrate the standardization method.

DNA yield in 50 µL was quantified using a Qubit 4 Fluorometer using the 1× dsDNA High Sensitivity Assay Kit (Life Technologies Corp., Carlsbad, CA, USA). To account for differences in beetle size, DNA yield was adjusted to obtain the expected yield given a starting length of 2000 µm $\left[\frac{\mathrm{yield}\;\left(\mathrm{ng}\right)}{\mathrm{beetle}\;\mathrm{length}\;\left(\mu m\right)}*2000\;\mu m\right]$ (Figure 4). Relative efficiency of each treatment was determined by comparing standardized DNA yield using analysis of variance (SAS 9.3).

### 4.3. DNA Quality

Downstream applications of DNA such as amplification and sequencing can be negatively affected by fragmentation that occurs during the extraction process and leftover inhibitor compounds. Although fragmentation can be estimated using gel electrophoresis of extracted DNA based on band intensity, size, and amount of DNA smearing [23], the very low quantities of DNA obtained from Scolytinae beetles makes this approach impractical. To compare differences in overall DNA fragmentation and quality among treatments, a PCR was conducted on all samples using primers D2F1 and D3R2 [18].

Reactions were performed in 25 μL volumes, consisting of 12.5 μL 2× Immomix Red (Bioline), 1 μL each of 10 μm forward and reverse primer, 1.5 ng DNA template, and sterile nuclease free water to 25 μL. Touchdown PCR’s were performed on a Bio-Rad C1000 Touch thermal cycler (Hercules, CA, USA) using a program developed during this study (Table 8).

Amplified products were electrophoresed on 1% (w/v) agarose gels containing 8 μL of Biotium GelRed (Fremont, CA, USA) and visualized using Syngene InGenius Gel Imaging System (Frederick, MD, USA) and GeneSnap software version 7.08. PCR products were purified using the Qiagen PCR Purification Kit (Hilden, Germany) and concentrations were obtained as described above. DNA quality was quantified as the concentration of amplified product, and mean concentration of amplified product was compared among treatments using analysis of variance (SAS 6.3).

### 4.4. DNA Threshold for Molecular Identification Using D2F1/D3R2

To determine the lowest amount of DNA that can be used for molecular identification of ambrosia and bark beetles using D2F1/D3R2, PCRs were performed using seven progressively smaller quantities of DNA (1.5, 0.3, 0.06, 0.012, 0.0024, 0.00048, and 0.000096 ng), decreasing by a factor of five each time. For each level, amplification was attempted from DNA of three specimens (*Theoborus* sp.) and one negative control (water) using the primer pair D2F1/D2R2 (Table S2). Amplification reactions and PCR product visualization was carried out as described above.

For levels where clear bands were obtained from all samples (1.5 and 0.3 ng), one representative of each was sequenced to serve as a positive control against which to compare the accuracy of the sequences obtained at lower DNA thresholds (and lower concentration PCR product). At the lower concentrations, samples showing bands of any intensity were purified and sequenced to confirm amplification success and the quality of resulting sequences.

### 4.5. Primer Pairs for Molecular Identification of Diverse Genera

To identify primer pairs most useful for identifying a range of ambrosia and bark beetles, five primer pairs were selected from the literature based on successful amplification and sequencing of ambrosia and bark beetle DNA: D2F1 and D3R2 [18], LCO1490 and HCO2198 [19], eflafor1 and eflarev1 [20], ets149 and efa754 [21], and ITS2F and ITS2R [22] (Table 1). DNA was extracted using the N10 protocol, and each genus was represented by one to five beetles, depending upon availability (5 *Coptoborus*, 1 *Xyleborus*, 4 *Theoborus*, 4 *Hypothenemus*, 3 *Araptus*). Primers were assessed based on their ability to amplify samples in each genus, their ability to generate high-quality sequences, and the ability of these sequences to provide correct identification following BLASTn analysis.

## 5. Conclusions

A wide variety of Scolytinae can currently be identified to the genus level using genetic sequences. Based on the results from this study, rapid DNA extraction (N10) is recommended due to the shorter time required. Molecular identification can be done even when very little DNA is recovered, as 0.3 ng DNA is sufficient for reliable amplification and sequencing with primer pair D2F1/D3R2 using the program described here. DNA yields lower than this are not necessarily an impediment, however, correct identification was obtained in this study using as little as 0.0024 ng.

Markers COI and EF-1a, specifically primer pairs LCO1490/HCO2198 and eflafor1/eflarev1, are recommended based on the ability of the respective primers to amplify DNA from multiple genera (*Coptoborus*, *Xyleborus*, *Theoborus*, *Hypothenemus*, and *Araptus*), and the accurate and unambiguous matches found in GenBank for the resulting sequences. If satisfactory results are not obtained with these markers, 28S (primer pair D2F1/D3R2) can be used as an alternative. Primers D2F1 and D3R2 amplified the greatest number of individuals in all groups and correctly identified the ambrosia beetle genera (*Coptoborus*, *Xyleborus*, *Theoborus*). However, the 28S sequences generated from the bark beetles, *Hypothenemus* and *Araptus*, did not provide accurate identification, likely due to low representation in GenBank. As more sequences are deposited in GenBank, the resolution and number of organisms that can be identified using molecular means are expected to increase.

## Figures and Tables

**Table 1:** Genes, amplicon size, and primer sequences for each primer pair used in this study. Reactions were performed using touchdown programs optimized for each primer pair. Amplified products were visualized, purified, and sequenced as described previously.

Gene	Primer	Sequence (5′-3′)	Amplicon Size (bp)	Reference
28S	D2F1	ACTGTTGGCGACGATGTTCT	500–570	[18]
	D3R2	TCTTCGCCCCTATACCC		
COI	LCO1490	GGTCAACAAATCATAAAGATATTGG	630–650	[19]
	HCO2198	TAAACTTCAGGGTGACCAAAAAATCA		
EF-1a	eflafor1	TACGTAACCATCATTGATGCTYCC	500	[20]
	eflarev1	CTTCTTTACGTTCAATGGACCATCC		
	ets149	ATCGAGAAGTICGAGAAGGAGGCYCARGAAATGGG	585	[21]
	efa754	CCACCAATTTTGTAGACATC		
ITS	ITS2F	GTGGATCCTGTGAACTGCAGGACACATG	460	[22]
	ITS2R	GTGAATTCATGCTTAAATTTAGGGGGTA		

**Table 2:** Proportion of amplification, generation of quality sequences, and positive identification in members of five Scolytinae genera using different primer pairs. Amplification is based on visible bands at 150 ms exposure and at least 2 ng/μL. Generation of quality sequences is defined as sequences of ≥150 bp and a quality score ≥ 80% as determined by the Geneious Prime software (Ver 2019.2.1.). Identification is whether a BLASTn search gave correct matches to the genus level.

Primer Pair	Variable Assessed	*Xyleborus*	*Coptoborus*	*Theoborus*	*Hypothenemus*	*Araptus*
D2F1 D3R2	Amplification	1/1	5/5	4/4	3/4	3/3
	Quality	1/1	5/5	4/4	3/4	3/3
	Identification	1/1	5/5	4/4	0/4	0/3
eflafor1 eflarev1	Amplification	1/1	4/5	4/4	3/4	2/3
	Quality	1/1	4/5	4/4	2/4	2/3
	Identification	1/1	4/5	4/4	3/4	0/3
ets149 efa754	Amplification	1/1	4/5	4/4	3/4	3/3
	Quality	1/1	3/5	3/4	2/4	0/3
	Identification	1/1	4/5	3/4	2/4	0/3
ITS2F ITS2R	Amplification	1/1	3/5	0/4	0/4	0/3
	Quality	1/1	3/5	0/4	0/4	0/3
	Identification	0/1	0/5	0/4	0/4	0/3
LCO1490 HCO2198	Amplification	1/1	5/5	2/4	4/4	3/3
	Quality	1/1	2/5	1/4	0/4	2/3
	Identification	1/1	2/5	2/4	1/4	2/3

**Table 3:** BLASTn results for 28S sequences amplified and sequenced with primers D2F1 and D3R2. Concentration of PCR product sent for sequencing also shown to the left of the GenBank match.

28S (D2F1/D3R2)					
Sample	Genus	PCR Concentration (ng/μL)	GenBank Match	Accession Number	% Identical
B2	*Coptoborus pseudotenuis*	44.6	*Coptoborus pseudotenuis*	HM099689	100.0%
B4	*C. pseudotenuis*	45	*Coptoborus pseudotenuis*	HM099689	100.0%
B6	*C. pseudotenuis*	16.6	*Coptoborus pseudotenuis*	HM099689	100.0%
B7a	*C. pseudotenuis*	46.5	*Coptoborus pseudotenuis*	HM099689	100.0%
B7b	*C. pseudotenuis*	38.2	*Coptoborus pseudotenuis*	HM099689	100.0%
B3	*Xyleborus ferrugineus*	42.4	*Xyleborus volvulus*	HM099763	100.0%
			*X. perforans*	HM099747	100.0%
			*X. bispinatus*	HM099741	100.0%
			*X. affinis*	GU808581	100.0%
B14	*Theoborus* sp.	39.2	*Theoborus* sp.	HM099718	99.7%
BC1	*Theoborus* sp.	23	*Theoborus* sp.	HM099718	99.7%
BC9	*Theoborus* sp.	42.8	*Theoborus* sp.	HM099718	99.7%
BC13	*Theoborus* sp.	37.7	*Theoborus* sp.	HM099718	100.0%
B16	*Hypothenemus* sp.	2.31	*Theoborus* sp.	HM099718	99.7%
B17	*Hypothenemus* sp.	6.07	*Theoborus* sp.	HM099718	99.7%
B19	*Hypothenemus* sp.	4.74	*Theoborus* sp.	HM099718	99.7%
B20	*Hypothenemus* sp.		No amplification		
J1	*Araptus* sp.	4.91	*Ips duplicatus*	JX263733	93.2%
			*Araptus* sp.	AF375297	87.9%
J2	*Araptus* sp.	9.32	*Ips duplicatus*	JX263733	91.4%
			*Araptus* sp.	AF375297	87.0%
J4	*Araptus* sp.	8.08	*Ips duplicatus.*	JX263733	91.5%
			*Araptus* sp.	AF375297	87.0%

**Table 4:** BLASTn results for EF-1a sequences amplified and sequenced with primers eflafor1 and eflarev1. Concentration of PCR product sent for sequencing also shown to the left of the GenBank match.

EF-1a (eflafor1/eflarev1)					
Sample	Genus	PCR Concentration (ng/μL)	GenBank Match	Accession Number	% Identical
B2	*Coptoborus pseudotenuis*	4.37	*Coptoborus pseudotenuis*	AF508880	99.7%
B4	*C. pseudotenuis*	4.36	*Coptoborus pseudotenuis*	AF508880	99.7%
B6	*C. pseudotenuis*		No amplification		
B7a	*C. pseudotenuis*	11	*Coptoborus pseudotenuis*	AF508880	100.0%
B7b	*C. pseudotenuis*	5.1	*Coptoborus pseudotenuis*	AF508880	100.0%
B3	*Xyleborus ferrugineus*	2.92	*Xyleborus ferrugineus*	KP941383	100.0%
B14	*Theoborus* sp. *Theoborus* sp.	15.8	*Theoborus* sp.	HM064194	99.6%
			*T. theobromae*	AF259881	99.6%
BC1	*Theoborus* sp.	10.2	*Theoborus* sp.	HM064194	99.7%
BC9	*Theoborus* sp.	14.1	*Theoborus* sp.	HM064194	99.6%
BC13	*Theoborus* sp.	19	*Theoborus* sp.	HM064194	99.7%
B16	*Hypothenemus* sp.	10.3	*Hypothenemus nr. eruditus*	JX264092	97.0%
B17	*Hypothenemus* sp.		No amplification		
B19	*Hypothenemus* sp.	16.1	*H. nr. eruditus*	JX264092	97.7%
B20	*Hypothenemus* sp.	5	*Hypothenemus* sp.	AF186658	96.0%
			*H. nr. eruditus*	JX264092	96.0%
J1	*Araptus* sp.		No amplification		
J2	*Araptus* sp.	11.2	*Phelloterus* sp.	KY805860	91.6%
J4	*Araptus* sp.	7.36	*Phelloterus* sp.	KY805860	91.6%
			*Araptus attenuatus*	FJ347565	89.6%

**Table 5:** BLASTn results for EF-1a sequences amplified and sequenced with primers ets149 and efa754. Concentration of PCR product sent for sequencing also shown to the left of the GenBank match.

EF-1a (ets149/efa754)					
Sample	Genus	PCR Concentration (ng/μL)	GenBank Match	Accession Number	% Identical
B2	*Coptoborus pseudotenuis*	12.9	*Coptoborus pseudotenuis*	AF508880	99.7%
B4	*C. pseudotenuis*	12.4	*Coptoborus pseudotenuis*	AF508880	99.0%
B6	*C. pseudotenuis*		No amplification		
B7a	*C. pseudotenuis*	16.3	*Coptoborus pseudotenuis*	AF508880	100.0%
B7b	*C. pseudotenuis*	5.1	*Coptoborus pseudotenuis*	AF508880	100.0%
B3	*Xyleborus ferrugineus*	38.9	*Xyleborus ferrugineus*	KP941383	94.7%
B14	*Theoborus* sp.	9.53	*Theoborus theobromae*	AF259881	99.6%
BC1	*Theoborus* sp.	10.2	*Theoborus* sp.	HM064194	99.7%
BC9	*Theoborus* sp.	5.56	Poor sequence		
BC13	*Theoborus* sp.	9.05	*Theoborus theobromae*	AF259881	100.0%
B16	*Hypothenemus* sp.	25.9	*Hypothenemus nr. eruditus*	JX264092	98.1%
B17	*Hypothenemus* sp.		No amplification		
B19	*Hypothenemus* sp.	25.9	*Hypothenemus nr. eruditus*	JX264092	99.3%
B20	*Hypothenemus* sp.	20.4	Poor sequence		
J1	*Araptus* sp.	7.62	Poor sequence		
J2	*Araptus* sp.	38.2	Poor sequence		
J4	*Araptus* sp.	14.9	Poor sequence		

**Table 6:** BLASTn results for ITS2 sequences amplified and sequenced with primers ITS2F and ITS2R. Concentration of PCR product sent for sequencing also shown to the left of the GenBank match. No match was determined by a query coverage > 30%.

ITS2 (ITS2F/ITS2R)					
Sample	Genus	PCR Concentration (ng/μL)	GenBank Match	Accession Number	% Identical
B2	*Coptoborus pseudotenuis*	12.70	No match		
B4	*C. pseudotenuis*		No amplification		
B6	*C. pseudotenuis*		No amplification		
B7a	*C. pseudotenuis*	25.80	No match		
B7b	*C. pseudotenuis*	12.10	*Pityogenes chalcographus*	JQ066311	100.0%
			*Galapaganus* spp.	EU748796	100.0%
B3	*Xyleborus ferrugineus*	21.00	*Pityogenes chalcographus*	JQ066311	100.0%
			*Galapaganus* spp.	EU748796	100.0%
B14	*Theoborus* sp.		No amplification		
BC1	*Theoborus* sp.		No amplification		
BC9	*Theoborus* sp.		No amplification		
BC13	*Theoborus* sp.		No amplification		
B16	*Hypothenemus* sp.		No amplification		
B17	*Hypothenemus* sp.		No amplification		
B19	*Hypothenemus* sp.		No amplification		
B20	*Hypothenemus* sp.		No amplification		
J1	*Araptus* sp.		No amplification		
J2	*Araptus* sp.		No amplification		
J4	*Araptus* sp.		No amplification		

**Table 7:** BLASTn results for COI sequences amplified and sequenced with primers LCO1490 and HCO2198. Concentration of PCR product sent for sequencing also shown to the left of the GenBank match.

COI (LCO1490/HCO2198)					
Sample	Genus	PCR Concentration (ng/μL)	GenBank Match	Accession Number	% Identical
B2	*Coptoborus pseudotenuis*	23.2	Poor sequence		
B4	*C. pseudotenuis*	12	Poor sequence		
B6	*C. pseudotenuis*	7.75	Poor sequence		
B7a	*C. pseudotenuis*	12.7	*Coptodyras* sp.	HM064072	86.4%
			*C. pseudotenuis*	HM064071	85.5%
B7b	*C. pseudotenuis*	21.8	*Coptodyras* sp.	HM064072	84.7%
			*C. pseudotenuis*	HM064071	84.3%
B3	*Xyleborus ferrugineus*	60	*Xyleborus ferrugineus*	KP941251	98.0%
B14	*Theoborus* sp.		No amplification		
BC1	*Theoborus* sp.		No amplification		
BC9	*Theoborus* sp.	5.87	*Theoborus* sp.	HM064100	94.7%
BC13	*Theoborus* sp.	38.9	*Theoborus* sp.	HM064100	91.2%
B16	*Hypothenemus* sp.	51	Poor sequence		
B17	*Hypothenemus* sp.	6.26	Poor sequence		
B19	*Hypothenemus* sp.	38.5	Poor sequence		
B20	*Hypothenemus* sp.	21.3	*Hypothenemus* sp.	MK768187	97.8%
			*Hypothenemus* sp.	KX035186	97.8%
J1	*Araptus* sp.	6.45	Poor sequence		
J2	*Araptus* sp.	8.38	*Araptus carinifrons*	MK768387	84.1%
J4	*Araptus* sp.	7.25	*Araptus carinifrons*	MK767301	84.4%

**Table 8:** Thermal cycler programs were optimized for each primer pair. All reactions started at 95 °C for 12 min (Immomix), followed by 94 °C for 1 min in primer-specific annealing conditions, and an extension at 72 °C for 1 min. A final elongation at 72 °C for 5 min was used in all reactions. Primer-specific conditions are shown here.

Gene	Primer	Annealing Temp (°C)	Annealing Time (s)	Touch Down Temp (°C)/cycle	Number of Cycles
28S	D2F1	58	45	−0.1	33
	D3R2				
COI	LCO1490	51	45	−0.2	40
	HCO2198				
EF-1a	eflafor1	56	45	−0.1	38
	eflarev1				
	ets149	51	45	−0.1	40
	efa754				
ITS	ITS2F	56	30	−0.1	40
	ITS2R				

## Supplementary Materials

Supplementary materials can be found at https://www.mdpi.com/1422-0067/20/23/5944/s1.

## References

[1] Hulcr J., Dunn R.R. (2011). The sudden emergence of pathogenicity in insect-fungus symbioses threatens naive forest ecosystems. Proc. R. Soc..

[2] Ploetz R.C., Hulcr J., Wingfield M.J., de Beer Z.W. (2013). Destructive Tree Diseases Associated with Ambrosia and Bark Beetles: Black Swan Events in Tree Pathology?. Plant Dis..

[3] Lee J.C., Haack R.A., Negron J.F., Witcosky J.J., Seybold S.J. Invasive Bark Beetles. http://purl.access.gpo.gov/GPO/LPS90197.

[4] Aukema J.E., McCullough D.G., Von Holle B., Liebhold A.M., Britton K., Frankel S.J. (2010). Historical Accumulation of Nonindigenous Forest Pests in the Continental United States. Bioscience.

[5] Webber J. (1981). A Natural Biological-Control of Dutch Elm Disease. Nature.

[6] Hanula J.L., Mayfield A.E., Fraedrich S.W., Rabaglia R.J. (2008). Biology and host associations of redbay ambrosia beetle (Coleoptera: Curculionidae: Scolytinae), exotic vector of laurel wilt killing redbay trees in the southeastern United States. J. Econ. Entomol..

[7] Kendra P.E., Montgomery W.S., Niogret J., Pruett G.E., Mayfield A.E., MacKenzie M., Deyrup M.A., Bauchan G.R., Ploetz R.C., Epsky N.D. (2014). North American Lauraceae: Terpenoid emissions, relative attraction and boring preferences of redbay ambrosia beetle, Xyleborus glabratus (Coleoptera: Curculionidae: Scolytinae). PLoS ONE.

[8] Tisserat N., Cranshaw W., Leatherman D., Utley C., Alexander K. (2009). Black walnut mortality in Colorado caused by the walnut twig beetle and thousand cankers disease. Phytopathology.

[9] Eskalen A., Gonzalez A., Wang D.H., Twizeyimana M., Mayorquin J.S., Lynch S.C. (2012). First Report of a Fusarium sp. and Its Vector Tea Shot Hole Borer (Euwallacea fornicatus) Causing Fusarium Dieback on Avocado in California. Plant Dis..

[10] Kendra P.E., Owens D., Montgomery W.S., Narvaez T.I., Bauchan G.R., Schnell E.Q., Tabanca N., Carrillo D. (2017). α-Copaene is an attractant, synergistic with quercivorol, for improved detection of Euwallacea nr. fornicatus (Coleoptera: Curculionidae: Scolytinae). PLoS ONE.

[11] Haack R.A. (2016). Exotic bark- and wood-boring Coleoptera in the United States: Recent establishments and interceptions. Can. J. For. Res..

[12] Haack R.A., Cavey J.F. (1997). Insects Intercepted on Wood Articles at ports-of-Entry in the United States: 1985-1996. Newsl. Mich. Entomol. Soc..

[13] Haack R.A., Cavey J.F. Insects Intercepted on Solid Wood Packing Materials at United States Ports-of-Entry: 1985–1998. https://www.nrs.fs.fed.us/pubs/2116.

[14] Haack R.A. (2001). Intercepted Scolytidae (Coleoptera) at U.S. ports of entry: 1985–2000. Integr. Pest Manag. Rev..

[15] Cognato A.I., Sperling F.A.H. (2000). Phylogeny of Ips DeGeer species (Coleoptera: Scolytidae) inferred from mitochondrial cytochrome oxidase I DNA sequence. Mol. Phylog. Evol..

[16] Jordal B.H., Sequeira A.S., Cognato A.I. (2011). The age and phylogeny of wood boring weevils and the origin of subsociality. Mol. Phylog. Evol..

[17] O’Donnell K., Libeskind-HadasJiri R. (2016). Invasive Asian Fusarium – Euwallacea ambrosia beetle mutualists pose a serious threat to forests, urban landscapes and the avocado industry. Phytoparasitica.

[18] Jordal B.H., Gillespie J.J., Cognato A.I. (2008). Secondary structure alignment and direct optimization of 28S rDNA sequences provide limited phylogenetic resolution in bark and ambrosia beetles (Curculionidae: Scolytinae). Zool. Scr..

[19] Hebert P.D., Cywinska A., Ball S.L., deWaard J.R. (2003). Biological identifications through DNA barcodes. Proc. Biol. Sci..

[20] Dole S.A., Jordal B.H., Cognato A.I. (2010). Polyphyly of Xylosandrus reitter inferred from nuclear and mitochondrial genes (Coleoptera: Curculionidae: Scolytinae). Mol. Phylog. Evol..

[21] Normark B.B., Jordal B.H., Farrell B.D. (1999). Origin of a haplodiploid beetle lineage. Proc. Biol. Soc..

[22] Andreev D., Breilid H., Kirkendall L., Brun L.O., Ffrench-Constant R.H. (1998). Lack of nucleotide variability in a beetle pest with extreme inbreeding. Insect Mol. Biol..

[23] Georgiou C.D., Papapostolou I. (2006). Assay for the quantification of intact/fragmented genomic DNA. Anal. Biochem..

[24] Sim S.B., Yoneishi N.M., Brill E., Geib S.M., Follett P.A. (2016). Molecular Markers Detect Cryptic Predation on Coffee Berry Borer (Coleoptera: Curculionidae) by Silvanid and Laemophloeid Flat Bark Beetles (Coleoptera: Silvanidae, Laemophloeidae) in Coffee Beans. J. Econom. Entomol..

[25] Mitchell A., Maddox C. (2010). Bark beetles (Coleoptera: Curculionidae: Scolytinae) of importance to the Australian macadamia industry: An integrative taxonomic approach to species diagnostics. Aust. J. Entomol..

[26] Masood A., Stoeckle B.C., Kuehn R., Saeed S. (2011). Cross Species Transfer of Microsatellite Loci in Scolytidae Species Mostly Associated with Mango (Mangifera indica L.; Anacardiaceae) Quick Decline Disease. Pak. J. Zool..

[27] Kendra P.E., Sanchez J.S., Montgomery W.S., Okins K.E., Niogret J., Pena J.E., Epsky N.D., Heath R.R. (2011). Diversity of Scolytinae (Coleoptera: Curculionidae) Attracted to Avocado, Lychee, and Essential Oil Lures. Fla. Entomol..

[28] Cognato A.I., Sun J.H. (2007). DNA based cladograms augment the discovery of a new Ips species from China (Coleoptera: Curculionidae: Scolytinae). Cladistics.

[29] Marchal L., Cazeres S., Kergoat G.J., Letellier K., Mitchell A., Nattier R., Mille C. (2017). A new pest of lychees in New Caledonia. N. Z. J. Zool..

[30] Campbell P.R., Geering A.D.W. Biosecurity Capacity Building for the Australian Avocado Industry: *Laurel Wilt*. http://www.avocadosource.com/wac7/Section_03/CampbellPaul2011.pdf.

[31] Simon C., Frati F., Beckenbach A., Crespi B., Liu H., Flook P. (1994). Evolution, weighting, and phylogenetic utility of mitochondrial gene sequences and a compilation of conserved polymerase chain reaction primers. Ann. Entomol. Soc. Am..

